# Chorioamnionitis and neonatal outcome: early vs late preterm infants

**DOI:** 10.1186/1824-7288-40-S2-A22

**Published:** 2014-10-09

**Authors:** Lidia Decembrino, Margherita Pozzi, Rossana Falcone, Mauro Stronati

**Affiliations:** 1 Neonatal Intensive Care Unit, Fondazione IRCCS Policlinico San Matteo, Pavia, Italy

## 

Chorioamnionitis (CA) describes an intrauterine status of inflammation and/or infection of placental membranes, refering to both histological and clinical CA [1]. It is considered the major risk of spontaneous preterm delivery, especially at earlier gestational age. The intrauterine exposure to infection/inflammation leads to the fetal inflammatory syndrome (FIRS) that together with CA is responsible for multiple organ injury, neonatal morbidity and mortality [2]. Strong evidences support that neonates exposed to CA are sicker at birth, have a higher rates of early-onset sepsis, respiratory distress syndrome (RDS), bronchopulmonary dysplasia (BPD), intraventricular hemorrhage (IVH), retinopathy of prematurity (ROP), patent ductus arteriousus (PDA) and surgical necrotizing enterocolitis (NEC) as compared with unexposed neonates [3-6]. Neonates with ≤ 28 weeks of gestational age (GA) have a significantly higher mortality than neonates with a longer gestation period [7]. Recently Pappas et al reported an increased odds of cognitive impairment and death/neurodevelopmental impairment in extremely low birth weight (ELBW) exposed to CA [8]. In infants born at 36 weeks or later in gestation CA has been indentified as an independent risk factor of CP [9]. Lee et al highlighted that acute histologic CA is a risk factor for adverse neonatal outcome in late preterm birth after preterm premature rupture of membranes (PPROM) [10]. Neverthless, the effects of CA on the neonatal outcome remain under debate, because gestation-independent effects of CA on neonatal outcomes are difficult to assess. Thus in some studies at adjusted analyses for GA, the adverse impact of CA on neonatal outcome is not confirmed [11]. Additionally in many study groups discrimination between ELBW and late preterm infants is not considered. In the future, sufficiently powered cohort studies and well-matched case-control studies will be able to provide useful informations regarding the different outcome between extremely and late preterms infants. An dequate antenatal screening and treatment for CA will improve the prognosis for infants at risk of multiple organ disease as a result of exposure to infection/inflammation before birth [12-14].
